# Synthesis and Characterization of Poly(DL-lactide) Containing Fluorene Structures

**DOI:** 10.3390/polym15112555

**Published:** 2023-06-01

**Authors:** Chung-Fu Yu, Syang-Peng Rwei, Shung-Jim Yang, Wen-Chin Tsen, Li-Huei Lin

**Affiliations:** 1 Institute of Organic and Polymeric Materials, Research, National Taipei University of Technology, Taipei 106344, Taiwan; 2 Research and Development Center for Smart Textile Technology, Taipei 106344, Taiwan; 3 Department of Aeronautical and Opto-Mechatronic Engineering, Vanung University, Taoyuan 320313, Taiwan; 4 Graduate School of Fabric Technology Management, Lee-Ming Institute of Technology, New Taipei City 243083, Taiwan; 5 Department of Cosmetic Science, Vanung University, Taoyuan 320313, Taiwan

**Keywords:** Poly(DL-lactide), fluorene, bisphenol fluorene, refractive index

## Abstract

9,9-bis[4-(2-hydroxy-3-acryloyloxypropoxy)phenyl]fluorene (BPF) hydroxyl groups (-OH) were used as initiators in the ring-opening polymerization reaction with DL-lactide monomers at different molar ratios to synthesize a Poly(DL-lactide) polymer containing bisphenol fluorene structure and acrylate functional groups (DL-BPF). The polymer’s structure and molecular weight range were analyzed using NMR (^1^H, ^13^C) and gel permeation chromatography. DL-BPF was then subjected to photocrosslinking using the photoinitiator Omnirad 1173, resulting in the formation of an optically transparent crosslinked polymer. Characterization of the crosslinked polymer involved analyzing its gel content, refractive index, thermal stability (via differential scanning thermometry (DSC) and thermogravimetric analysis (TGA)), as well as conducting cytotoxicity tests. The crosslinked copolymer exhibited a maximum refractive index of 1.5276, a maximum glass transition temperature of 61.1 °C, and cell survival rates higher than 83% in the cytotoxicity tests.

## 1. Introduction

Polylactide (PLA) is a bio-based polyester known for its high biocompatibility and biodegradability. It possesses desirable mechanical properties, including a high elastic modulus, high tensile strength, and low elongation at break. PLA finds applications in various fields such as consumer goods, packaging, agriculture, and biomedicine, and it is currently gaining popularity [1,2,3,4]. However, PLA faces limitations in terms of brittleness, low toughness, poor heat resistance, slow crystallization rates, and hydrophobicity. To overcome these limitations, researchers have explored methods such as chemical modification, copolymerization, and polymer blending to enhance the crystallinity, thermal stability, mechanical properties, and hydrophobicity/hydrophilicity of PLA [5,6,7,8,9].

In general, the performance of polylactic acid (PLA) can be enhanced by functionalizing its end groups, enabling the utilization of specific end-functional compounds for polymerization. Branched structures of PLA possess numerous multifunctional groups, and the physical, chemical, and mechanical properties of PLA can be modified by altering its molecular structure. Control over the molecular branching structure can be achieved through various polymer synthesis methods and processing strategies [10,11,12].

The literature demonstrates an increasing interest in the synthesis of branched and star-shaped PLA. The transition from linear PLA to branched and star-shaped structures has implications for biocompatibility, thermal properties, mechanical properties, rheological properties, degradation characteristics, drug release rate, crystallization nucleation, and the hydrophilicity/hydrophobicity of the polymer [13,14,15,16]. While copolymerization and processing methods can introduce diverse functional groups into PLA, the selection of co-monomers can involve compounds with higher hydrophilicity or superior biocompatibility, such as lactide, glycolide, polyethylene glycol, and polycaprolactone, to enhance the biocompatibility of the modified polymer [17,18,19,20,21,22].

PLA can undergo crosslinking through various methods including chemical, ionizing radiation, and UV/visible light crosslinking [23,24]. Prior to crosslinking, PLA needs to be functionalized with double bonds at the chain terminals. This is often achieved by copolymerizing PLA with low-molecular-weight diols or polyfunctional alcohols, resulting in OH-terminated PLA chains with bifunctional or multifunctional properties. These PLA chains can then be polymerized to form linear, branched, or star-shaped copolymers [14,25,26,27]. Subsequently, the copolymers are functionalized with acrylate groups. Finally, the double-bond-functionalized PLA polymer is mixed with a photoinitiator and exposed to UV or visible light to initiate free-radical polymerization [28,29,30]. During photocrosslinking, PLA chains form a stereocomplex, which enhances resistance to hydrolysis, thermal stability, and mechanical integrity. This process can improve the mechanical properties, thermal stability, degradability, and enable the creation of 3D structures [31,32,33,34]. However, it is important to note that crosslinking reduces the material’s degradability. Therefore, careful regulation of the degradation cycle is necessary by adjusting reaction conditions and process parameters [35,36].

Fluorene is a distinctive bisphenyl structure that was first discovered in 1867 by Berthelot during the distillation of crude anthracene oil. It possesses a fluorene ring structure, also known as a cardo skeleton. Fluorenes exhibit remarkable thermal stability and have an extended conjugation system, resulting in a broad absorption band. These characteristics make fluorenes highly photoluminescent, displaying strong fluorescence and phosphorescence, as well as electroluminescence. 9,9-Bis(4-hydroxyphenyl)fluorene (BHPF) is a cardo bisphenol compound synthesized through the acid-catalyzed condensation of phenol and fluorenone [37,38]. Due to its unique structure, BHPF demonstrates exceptional heat resistance and transparency, along with a high refractive index. In recent years, BHPF has been modified with various functional groups such as alcohols, phenols, epoxies, acrylics, carboxylic acids, and amines. These modified derivatives can undergo addition or condensation reactions with other monomers, leading to the formation of diverse polymer materials with applications in optoelectronics, electronics, and automotive industries [39,40,41,42]. Previous studies conducted by various researchers have investigated the incorporation of bisphenol fluorene derivatives into PLA and explored their impact on the thermal properties of the resulting materials [43,44].

BPF (9,9-bis[4-(2-hydroxy-3-acryloyloxypropoxy)phenyl]fluorene) is a monomer that combines bisphenol fluorene structure with hydroxyl and acrylate functional groups. It exhibits heat resistance and high refractive index properties. The acrylate functional groups at both ends enable free radical polymerization reactions. Poly(DL-lactide) (PDLLA) has an irregular and fully amorphous structure. PDLLA is characterized by the absence of a melting temperature (*T*_m_), and its glass transition temperature (*T*_g_) typically ranges between 40 and 55 °C, depending on the molecular weight. Currently, research on PDLLA is mainly focused on biomedical applications [45].

In this study, our goal was to utilize the hydroxyl groups (-OH) of BPF as initiators for ring-opening polymerization (ROP) with DL-lactide monomers. By varying the molar ratios of DL-lactide and BPF, we aimed to synthesize low-molecular-weight DL-BPF polymers that contain both bisphenol fluorene structures and acrylate functional groups. In the second phase, the acrylate functional groups of DL-BPF polymers were subjected to UV light-induced photocrosslinking to investigate the thermal properties and refractive index of the crosslinked DL-BPF polymers. This research aims to provide insights for the potential industrial and optical applications of this material.

## 2. Experimental Section

### 2.1. Materials Chemicals

DL-Lactide (99.5%) was purchased from Green Square Materials Inc and used as the monomer in the ring-opening polymerization. Tin (II) 2-ethylhexanoate (95%) from Alfa Aesar^®^ was employed as polymerization catalyst, whereas 9,9-bis[4-(2-hydroxy-3-acryloyloxypropoxy)phenyl]fluorine (BPF,95%) initiators were supplied from S.M.S Technology Co., Ltd., Taipei Taiwan. Acetone (99%, Aldrich) was the solvent used for purification of the final product. 4-methoxyphenol (99%) from Shora was employed as an inhibitor. 2-Hydroxy-2-methyl-1-phenylpropanone (Omnirad 1173) from I.G.M was employed as photoinitiator. All the materials were used as received without further treatment.

### 2.2. Synthesis of DL-BPF

**Scheme 1:**
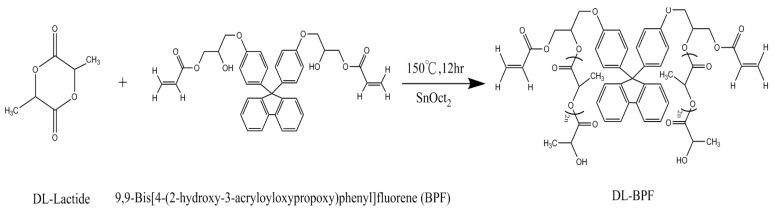
Synthesis of DL-BPF.

The DL-lactide and the 9,9-bis[4-(2-hydroxy-3-acryloyloxypropoxy)phenyl]fluorene (BPF) in molar ratios of 5:1, 10:1,15:1, 20:1, respectively, were placed in a 250 mL flask with three necks and dried under vacuum for 1 h. In a flask filled with dry nitrogen gas, Sn(Oct)_2_ (0.05 wt%) and 200 ppm 4-methoxyphenol were added. The mixture was heated at 150 °C using an oil bath for 12 h with stirring (Scheme 1). After the reaction, the reactor was cooled to room temperature. The resulting polymer was dissolved in acetone and then precipitated by adding it to an excess of deionized water while agitating the solution. The precipitated material was washed with deionized water and dried under reduced pressure [46,47,48].

### 2.3. Photocrosslinking Conditions

**Figure 1:**
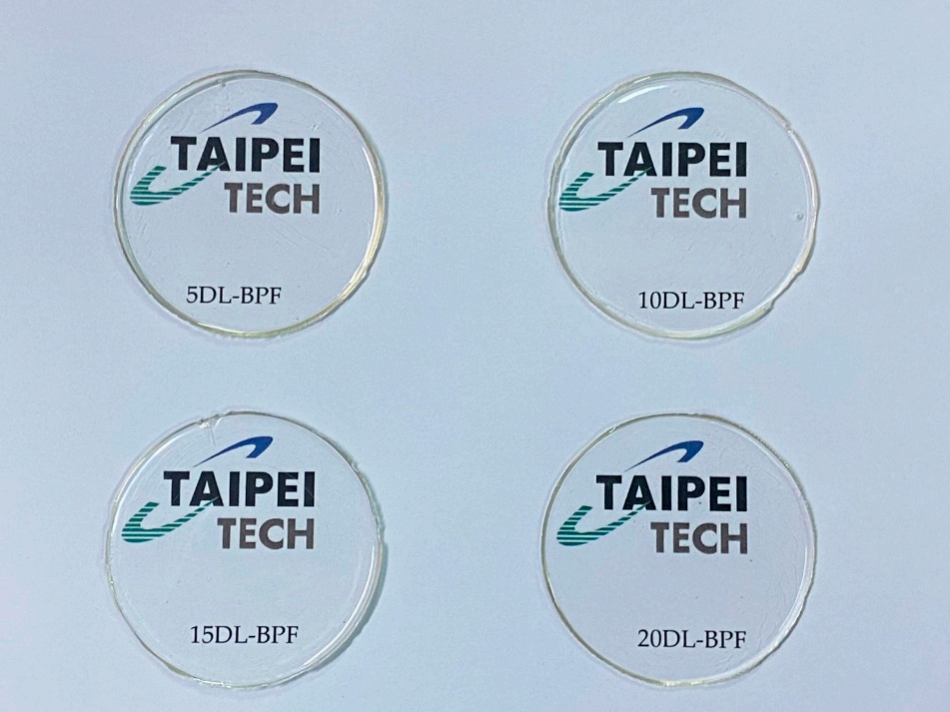
Photocrosslinking reactions were conducted using DL-BPF polymers with varying molar ratios.

Photocrosslinking was performed in a homemade UV vacuum oven (UV wavelength: 248–578 nm, intensity: 10–15 mW/cm^2^). The DL-BPF polymer was first dissolved in acetone, then mixed with the photoinitiator, Omnirad 1173 (3 wt%). After the solvent was removed, irradiation was performed under vacuum at 60 °C, for a maximum of 10 min irradiation, which yielded a transparent crosslinked polymer (Figure 1).

### 2.4. Polymer Characterization

#### 2.4.1. NMR Spectroscopy

The uncrosslinked DL-BPF polymer was dissolved using CDCl_3_ as the solvent. The chemical structure of DL-BPF was determined using Bruker Avance III HD-600 MHz NMR spectrometer to obtain ^13^C and ^1^H-NMR spectra.

#### 2.4.2. Gel Permeation Chromatography

Approximately 100–120 mg of uncrosslinked DL-BPF polymer was weighed and dissolved in tetrahydrofuran (THF). The molecular weight of the uncrosslinked DL-BPF polymer was measured using gel permeation chromatography (Jasco, RI-2031 Plus, Japan). The measurement conditions were as follows: the mobile phase was THF, the temperature was set at 35 °C, the flow rate of the mobile phase was 10 mL/min, and polystyrene was used as the calibration standard.

The polystyrene standard was obtained from POLYMER STANDARDS SERVICE (PSS). The GPC column setup consisted of three columns in a series: Jordi Gel DVB Mixed Bed, Waters Styragel^®^HR4E THF, and Waters Styragel^®^HR3 THF. The molecular weight was calculated based on the calibration curve obtained using the polystyrene standard, with an R-value of 0.9986.

#### 2.4.3. Differential Scanning Calorimetry (DSC)

The uncrosslinked and photocrosslinked DL-BPF polymer samples weighing approximately 5–8 mg were placed in dedicated aluminum pans for DSC (differential scanning calorimetry) analysis. The glass transition temperature (*T*_g_) of the polymer was measured using a HITACHI DSC7000X instrument, Japen. The measurement conditions were as follows: under a nitrogen atmosphere, heating rate of 10 °C/min, heating range from −10 °C to 110 °C, and the heating cycle was repeated twice. The glass transition temperature (*T*_g_) of the DL-BPF polymer was determined from the midpoint of the DSC curve obtained during the second heating cycle.

#### 2.4.4. Thermogravimetric Analysis (TGA)

The photocrosslinked DL-BPF polymer samples weighing between 4 and 6 mg were weighed and subjected to thermogravimetric analysis (TGA) using a Perkin–Elmer Pyris 1 TGA instrument. The measurement conditions were as follows: the samples were heated under a nitrogen flow of 20 cm^3^/min at a heating rate of 10 °C/min, reaching a temperature of 830 °C. The TGA analysis observed the thermal degradation behavior of the DL-BPF polymer during the heating process, tracking any changes in weight as a function of temperature.

#### 2.4.5. Attenuated Total Reflectance Fourier Transform Infrared Spectroscopy (ATR-FTIR)

The infrared spectra of DL-BPF polymer before and after photocrosslinking were measured using an attenuated total reflection Fourier transform infrared spectrometer (ATR-FTIR) equipped with a germanium crystal (PerkinElmer, Spectrum 100, USA). The instrument was set to scan the wavelength range of 550–4000 cm^−1^ with a resolution of 4 cm^−1^. Each sample was scanned 16 times to ensure reliable measurement results. To confirm the repeatability of the measurements, at least two measurements were performed for each sample.

#### 2.4.6. Derivation of Gel Content

Briefly, 400 mg of photocrosslinked DL-BPF polymer is mixed with 4 g of acetone, ensuring complete infiltration of the polymer by acetone. The mixture is left to stand at room temperature for 24 h. After 24 h, the solution containing the polymer is filtered to separate the insoluble polymer. The insoluble polymer is then placed in a vacuum oven at 50 °C and subjected to vacuum-drying for 12 h to ensure complete removal of acetone. The gel residue of DL-BPF polymer, which is insoluble in acetone, can be calculated using the following formula:

Gel residue (%) = (W_B_/W_A_) × 100%

W_A_: Initial weight of photocrosslinked DL-BPF polymer

W_B_: Weight of photocrosslinked DL-BPF polymer after soaking in acetone for 24 h, filtration, and vacuum-drying.

#### 2.4.7. Measurement of Refractive Index

A sample of photocrosslinked DL-BPF polymer with dimensions of 20 × 10 × 1 mm was prepared. The refractive index of the sample was measured using the ATAGO Abbe refractometer (DR-A1-Plus, ATAGO, Japan).

#### 2.4.8. Cytotoxicity Test

The cytotoxicity of photocrosslinked 10DL-BPF polymer was assessed according to the ISO 10993-5:2009 standard. Four sets of samples were prepared, including blank samples, negative control samples, positive control samples, and test samples. All of the tested materials were extracted using culture medium with 10% fetal bovine serum to prepare liquid extracts of the materials, which were then cultured with L929 mouse fibroblast cells for 24 h (at 5 ± 1% CO_2_ and 37 ± 1 °C). The cytotoxicity of the tested materials was qualitatively evaluated by monitoring the morphology of the L929 cells in accordance with the grading criteria of the ISO 10993-5:2009 standard, and the results were recorded. The MTT assay (cell viability analysis) was performed to determine the cell survival rates, using two-fold serial dilutions of the liquid extract (100, 50, 25, and 12.5%). The cells were incubated in these dilutions for 24 h in an incubator, with five replicate measurements for each dilution. All test materials were prepared according to the methods described in ISO 10993-12 standard.

## 3. Results

### 3.1. Spectral Characterization of ^1^H-NMR and ^13^C-NMR

**Figure 2:**
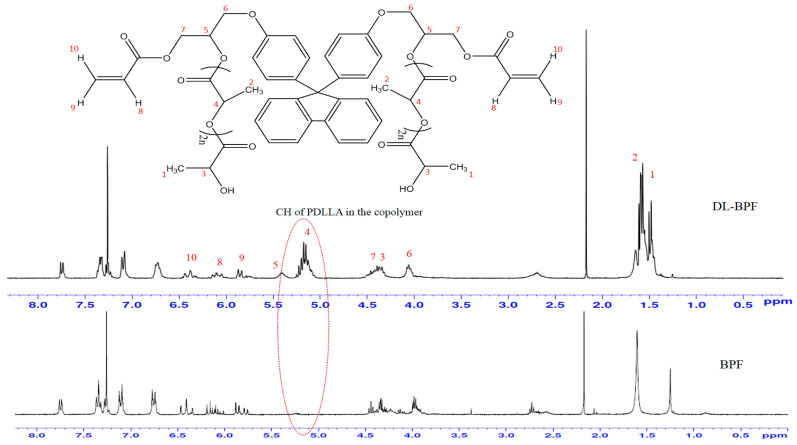
^1^H-NMR spectrum of DL-BPF before and after ring-opening polymerization.

Figure 2 shows the nuclear magnetic resonance (NMR) spectrum of DL-BPF. In the ^1^H-NMR spectrum, the -CH absorption peak of Poly(DL-lactide) was (δH4 = 5.14–5.23 ppm [^1^H, CH]), the methyl-CH_3_ absorption peak of Poly(DL-lactide) was (δH1, δH2 = 1.46–1. 61 ppm [^3^H, CH_3_]), the absorption peak of CH at one end of DL-BPF polymer was (δH3 = 4.31–4.39 ppm [^1^H, -CH]), the -CH absorption peak of BPF was (δH4 = 5.40–5. 48 ppm [^1^H, CH]), the CH_2_ absorption peak of BPF was (δH6 = 4.05–4.10 ppm, δH7 = 4.40–4.56 ppm [^2^H, CH_2_]), the vinyl hydrogen (-CH=CH_2_) absorption peak of BPF was (δH8, δH9, δH10 = 5. 75–6.42 ppm [^3^H, CH=CH_2_]), and the absorption of protons of benzene ring in BPF structure was (δH11, δH12, δH13, δH14, δH15, δH16 = 6.75–7.73 ppm [^1^H, CH]). The residual solvent peak is at 2.13 ppm. The polymers with DL-lactide and BPF structures were obtained by ring-opening polymerization as determined by ^1^H-NMR.

**Figure 3:**
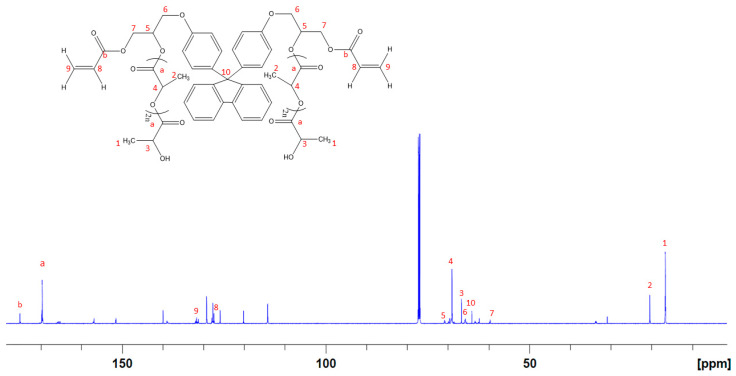
^13^C-NMR spectrum of DL-BPF.

In Figure 3, the peak at δ16.63, δ20.48 ppm is ascribed to the methyl group (CH_3_) carbon nuclei of Poly(DL-lactide) and the peaks in the range of δ66.70, δ69.01 ppm are the carbon nuclei of methane groups (CH). The peaks in the range of δ169.58 ppm are carboxyl carbon atom (C=O) carbon nuclei of Poly(DL-lactide). The peaks at δ59.63, δ65.79, δ131.24, δ131.69, δ131.99 ppm are ascribed to methylene groups’ (CH_2_) carbon nuclei of BPF and the peaks in the range of δ70.08, δ127.93, ppm are the carbon nuclei of methane groups (CH). The peaks in the range of δ175.07 ppm are carboxyl carbon atom (C=O) carbon nuclei of BPF.Carbon nuclei(10) of BPF are observed at δ64.12 ppm. The peaks in the range of δ114.42–δ156.86 ppm are the carbon nuclei of benzene ring. These data fully reveal the preparation of DL-BPF.

### 3.2. Gel Formation and Gel Permeation Chromatography of DL-BPF

**Figure 4:**
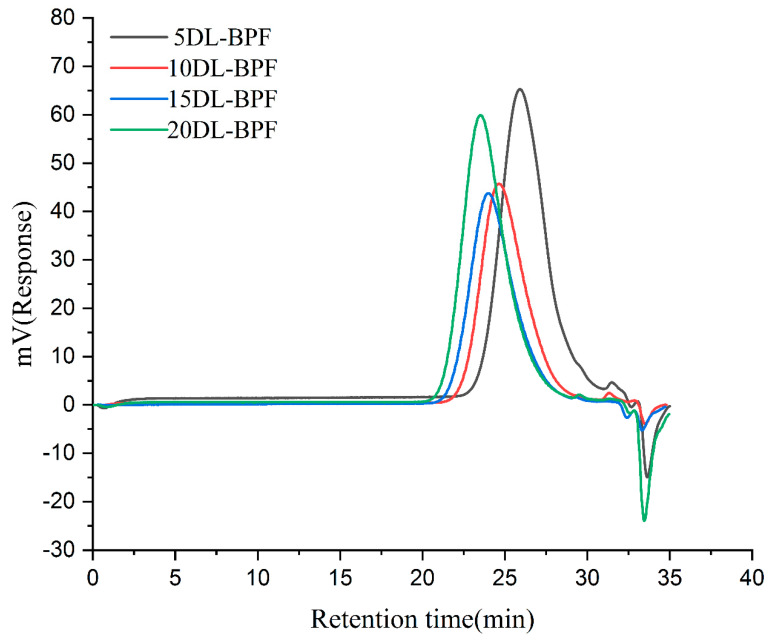
GPC diagrams of DL-BPF polymers, mobile phase: THF, 1 mL/min, 35 °C.

According to the GPC measurement results in Figure 4, DL-BPF exhibits a single peak, confirming its status as a single polymer. The molecular weight of DL-BPF polymers can be controlled by adjusting the molar ratio of DL-lactide to BPF. The GPC measurements indicate that the number-average molecular weight of DL-BPF polymers increases with higher DL-lactide molar ratios. Calculations based on the DL-lactide molecular weight (144 g/mol) multiplied by its molar ratio and adding the BPF molecular weight (606 g/mol) provide an estimated molecular weight range for DL-BPF, which closely aligns with the measured number-average molecular weight obtained from GPC.

Therefore, it can be inferred that DL-lactide undergoes ring-opening polymerization reactions with the alkyl groups on the BPF molecular chain, leading to the extension of the polymer chains. This process is controlled by the molar ratio of DL-lactide.

During the polymer crosslinking process, once the length of the polymer chains exceeds a certain threshold, glass transition occurs. This limits the participation of some free radicals and double bonds in the crosslinking reaction, preventing them from reaching 100% conversion. Consequently, the gel content test results demonstrate that DL-BPF polymers, regardless of their molar ratios and molecular weights, exhibit gel contents of 90% or higher (see Table 1).

**Figure 5:**
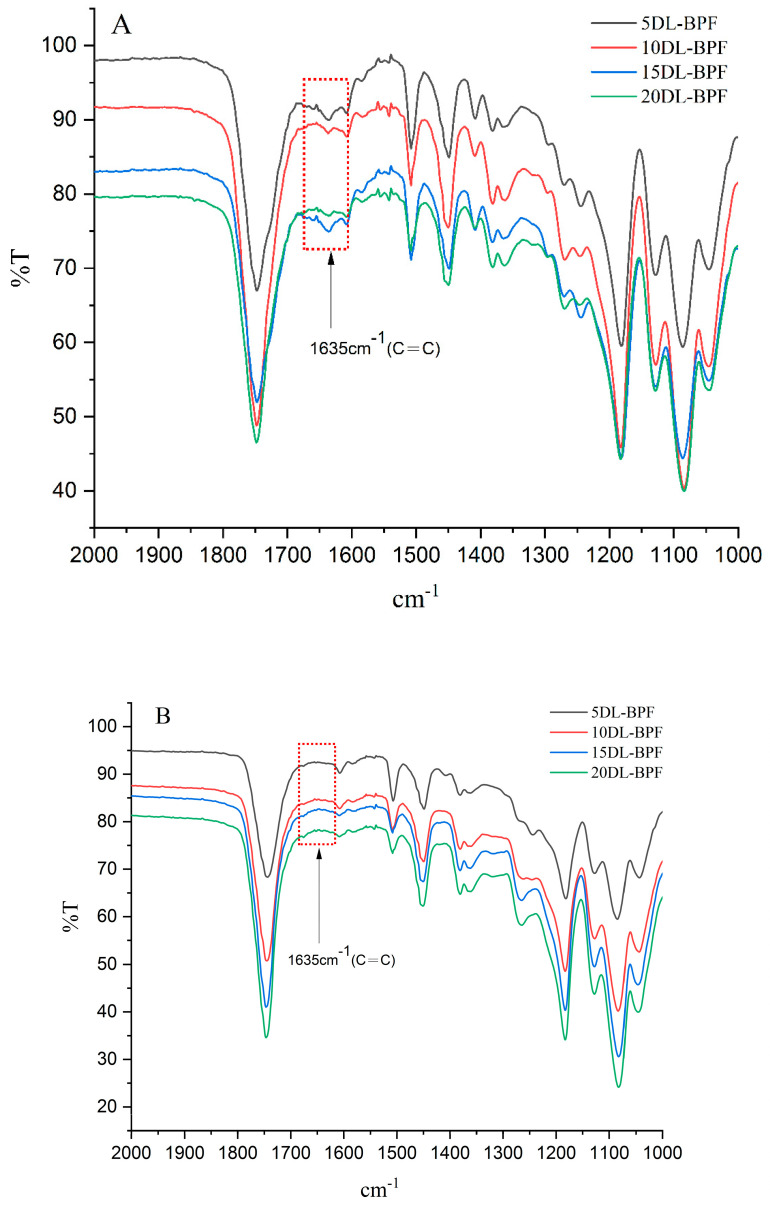
ATR-FTIR spectra of (**A**) pristine of DL-BPF (**B**) photocrosslinked of DL-BPF.

In the attenuated total reflection Fourier transform infrared (ATR-FTIR) spectrum shown in Figure 5, we observed the changes in the characteristic peak of the C=C double bond of the acrylate functional group in DL-BPF polymer. At the wavelength of 1635 cm^−1^, there is a distinct small peak, which represents the characteristic peak of the C=C double bond in the uncrosslinked DL-BPF polymer. After UV irradiation, the characteristic peak of the C=C double bond disappears, indicating that under the presence of the photoinitiator Omnirad 1173, the acrylate functional groups (C=C) at both ends of the DL-BPF polymer undergo free radical polymerization upon UV irradiation and form a crosslinked network structure in the DL-BPF polymer.

### 3.3. Refractive Index of Photocrosslinked DL-BPF

The measurement data in Table 2 show that the refractive index of DL-BPF polymers decreases with an increase in the molar ratio of DL-lactide monomers. BPF monomers, due to the presence of bisphenol fluorene structures, have a higher refractive index. According to previous literature, the refractive index of PLA is reported to be 1.4557 [49]. Therefore, incorporating the bisphenol fluorene structures of BPF monomers into the Poly(DL-lactide) chains can indeed increase the refractive index of DL-BPF polymers. Depending on the molar ratio, the refractive index of DL-BPF polymers can range from 1.4852 to 1.5276. However, it was also observed that the refractive index decreases as the molar ratio of DL-lactide monomers increases. This suggests that after the completion of the ring-opening polymerization reaction, the molecular chains of DL-BPF polymers also elongate. Upon photocrosslinking, the Poly(DL-lactide) segments occupy a larger proportion of the DL-BPF molecular chains, thereby limiting the increase in refractive index.

### 3.4. Thermal Analysis

**Figure 6:**
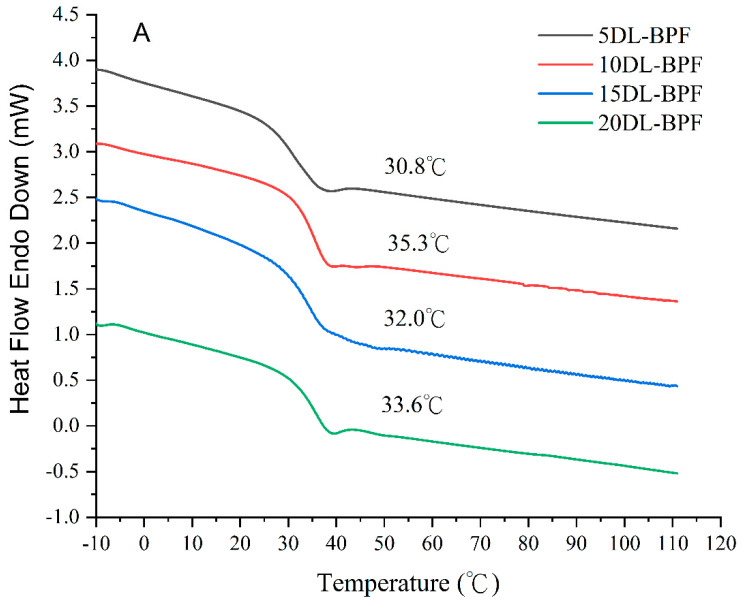

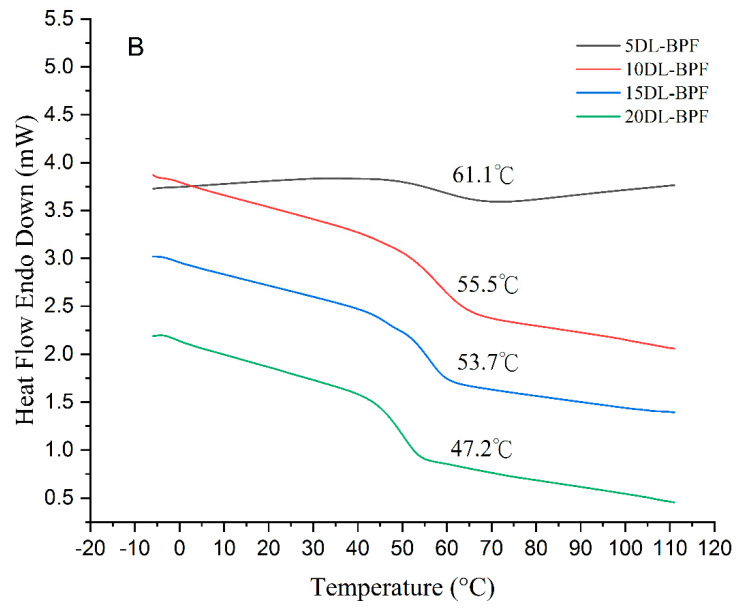
DSC thermograms of (**A**) pristine of DL-BPF (**B**) photocrosslinked of DL-BPF.

Table 3 presents the thermal coefficients of DL-BPF, which were determined through differential scanning calorimetry (DSC) and thermogravimetric analysis (TGA). The initial DL-BPF polymer, prior to photocrosslinking, consists of short chains. However, after undergoing photocrosslinking, it transforms into an optically transparent polymer with a network-like structure. This structural transformation leads to an increase in the glass transition temperature (*T*_g_) by 13.6–30.3 °C, as shown in Figure 6. The extent of this increase depends on the molar ratio of the reactants. The inherent thermal stability of the bisphenol fluorene structure in BPF contributes to the overall thermal stability of the polymer. The *T*_g_ and thermal degradation temperature of the DL-BPF polymer are influenced by factors such as chain structure, molecular weight, and degree of crosslinking. The densely crosslinked acrylate side chains and bisphenol fluorene structures in the polymer restrict the movement of polymer chains and reduce the free volume within the polymer matrix. Among the different DL-lactide-to-BPF molar ratios studied, a ratio of 5:1 was found to optimize the *T*_g_, resulting in a significant increase of 30.3 °C.

**Figure 7:**
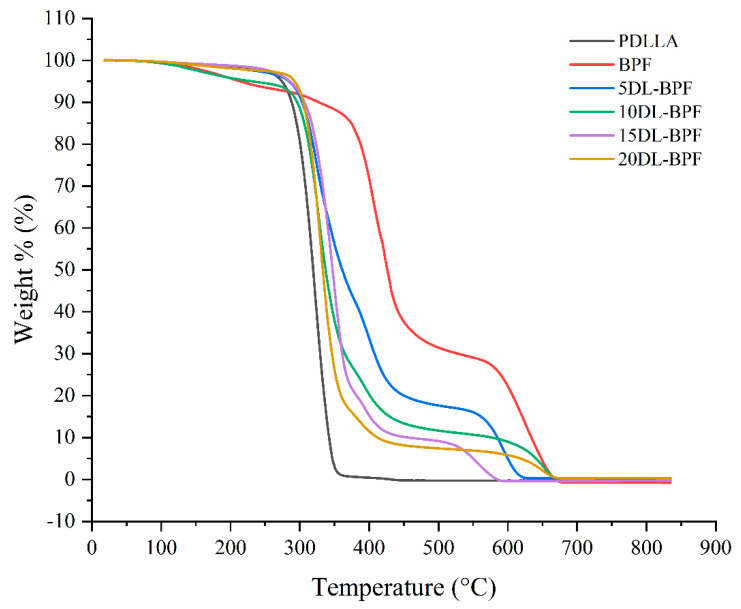

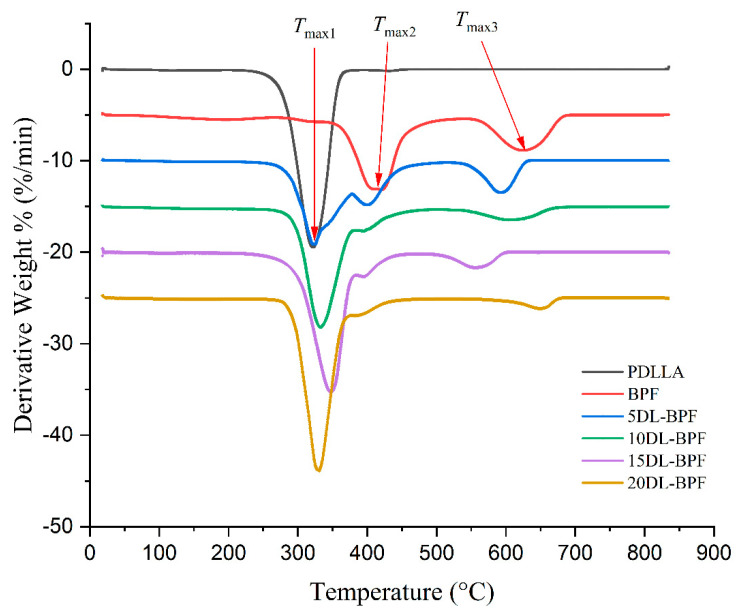
TGA/DTG thermograms of photocrosslinked DL-BPF.

As shown in Figure 7, the maximum thermal decomposition temperature of Poly(DL-lactide) is 319.37 °C. BPF exhibits a two-step thermal decomposition curve, with maximum decomposition temperatures at 420.95 °C and 626.0 °C. DL-BPF shows a three-step thermal decomposition curve, corresponding to the thermal decomposition curves of Poly(DL-lactide) and BPF. Additionally, we observed that as the molecular weight of DL-BPF polymer increased, the peak areas of BPF’s thermal decomposition (*T*_max2_ and *T*_max3_) decreased. This can be attributed to the proportion of BPF within the overall molecular chain, which decreases with an increase in the molar ratio of DL-lactide monomers.

Regarding the thermal decomposition temperature, although the DL-BPF polymer formed a crosslinked network structure after UV irradiation and contained bisphenol fluorene structures in its molecular chain, the thermal decomposition temperature did not show a significant increase. This suggests that the thermal properties of DL-BPF polymer, influenced by the heat resistance of bisphenol fluorene structures, were not significantly enhanced due to the predominant presence of Poly(DL-lactide) chains in the overall molecular structure. Consequently, the *T*_5%_ (temperature at which 5% weight loss occurs) of the photocrosslinked DL-BPF polymer and the thermal decomposition temperature of the Poly(DL-lactide) segments only slightly increased with an increase in the molecular weight.

### 3.5. The Cytotoxicity Testing of Photocrosslinked DL-BPF

**Figure 8:**
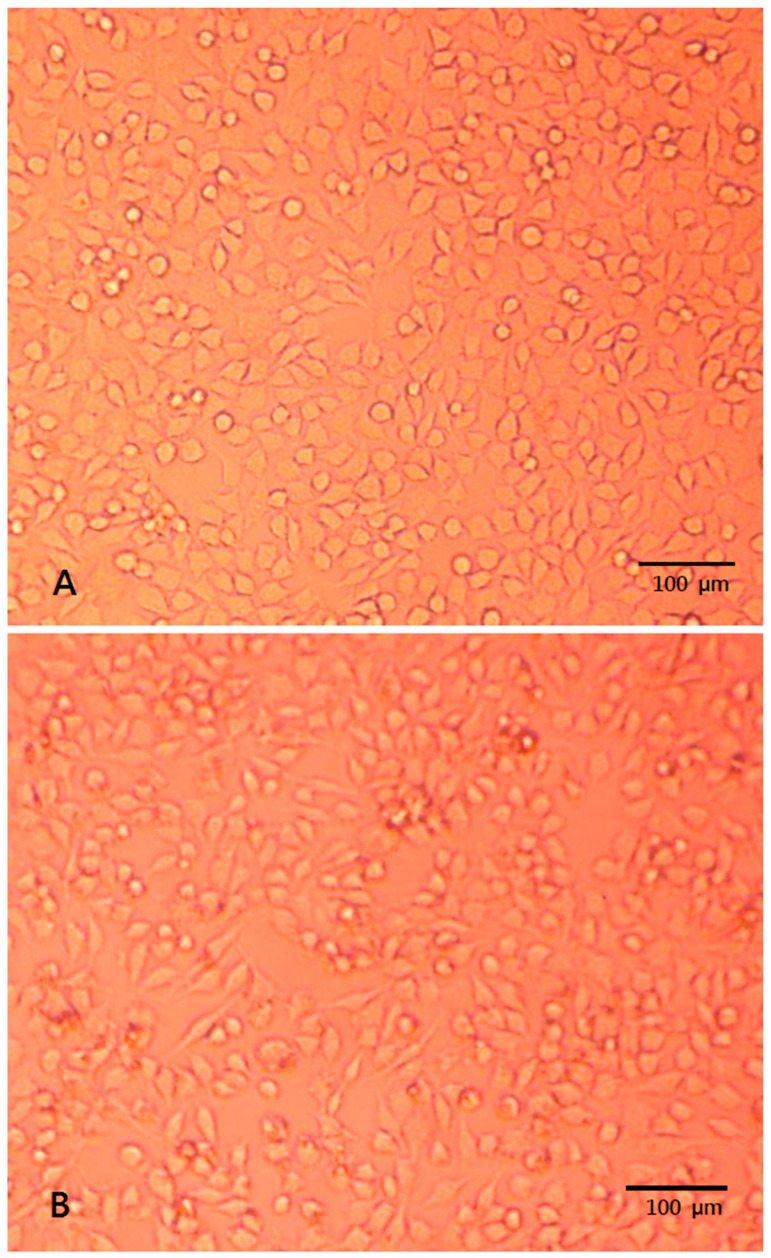
Morphology of the L929 mouse fibroblast cells after 24 h. (**A**) Cell morphologies in the blank group (cell culture medium that has undergone extraction), which are normal and show no signs of cell lysis. (**B**) Cell morphologies in the test group (with 10DL-BPF), which exhibit mild lysing and morphological changes.

**Figure 9:**
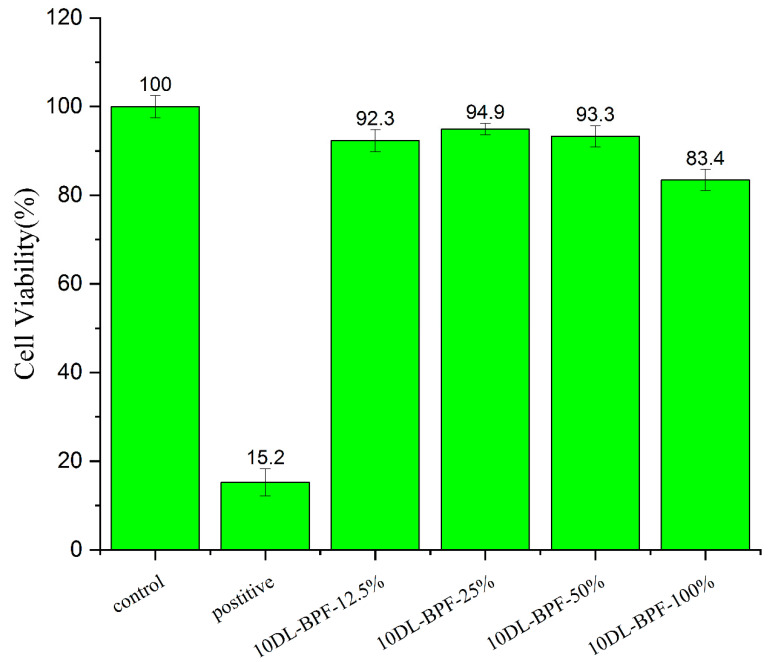
Bar graph of photocrosslinked 10DL-BPF cytotoxicity testing.

The cytotoxicity of photocrosslinked 10DL-BPF was evaluated according to the ISO 10993-5:2009 standard. An inverted microscope was used to observe the morphology of the L929 cells for qualitative morphological grading of the cytotoxicity. The cells exposed to 10DL-BPF were slightly lysed or altered, which corresponds to grade 1 cytotoxicity (see Figure 8). Quantitative evaluation of the cytotoxicity was performed by using the MTT assay to measure the cell survival rate, where the 100% 10DL-BPF extract contained 16.6% fewer cells than the control, corresponding to a cell survival rate of 83.4% (see Figure 9). According to the ISO 10993-5:2009 grading criteria, a material is deemed cytotoxic if it reduces the cell viability by more than 30% or achieves a numerical cytotoxicity grade greater than 2. Therefore, these results indicate that photocrosslinked 10DL-BPF is not cytotoxic to L929 cells.

## 4. Conclusions

In this study, we employed the -OH groups on the molecular chain of 9,9-bis[4-(2-hydroxy-3-acryloyloxypropoxy)phenyl]fluorene (BPF) as initiators and adjusted the molar ratios of DL-lactide monomers in the ring-opening polymerization reaction. By conducting analysis using ^13^C and ^1^H-NMR spectroscopy, we successfully synthesized low-molecular-weight polymers (DL-BPF) that contain both bisphenol fluorene structures and acrylate functional groups. Through careful control of the molar ratio of DL-lactide monomers, we achieved precise control over the molecular weight of DL-BPF within a specific range.

After the addition of the photoinitiator Omnirad 1173, the DL-BPF polymer, which contains acrylate functional groups at both ends, undergoes a free radical polymerization reaction initiated by UV light irradiation. The occurrence of photocrosslinking in the DL-BPF polymer was confirmed through ATR-FTIR spectroscopy, which showed a significant reduction and disappearance of the characteristic peak at 1635 cm^−1^ corresponding to the C=C double bond after UV light irradiation. This indicates the successful formation of crosslinks within the DL-BPF polymer.

Gel content measurements were performed to assess the degree of crosslinking in the DL-BPF polymer. The results revealed a gel content of 90%, indicating the formation of a highly crosslinked network structure when exposed to UV irradiation. This demonstrates the effectiveness of the photocrosslinking process in creating a stable and interconnected polymer network in the DL-BPF polymer.

After photocrosslinking, the DL-BPF polymer forms a tightly interconnected network structure due to its branched nature. The presence of bisphenol fluorene structures in the molecular chains restricts their rotational motion, resulting in an increased glass transition temperature (*T*_g_) of the DL-BPF polymer. The DSC analysis showed that the *T*_g_ can increase by up to 30.3 °C after photocrosslinking.

In terms of thermal decomposition analysis, although the DL-BPF polymer forms a network structure after photocrosslinking, the presence of Poly(DL-lactide) chains still occupies a significant portion of the overall molecular structure. Therefore, the thermal decomposition temperature of the DL-BPF polymer did not show a significant increase due to the formation of a crosslinked network and the presence of bisphenol fluorene structures.

Furthermore, the incorporation of bisphenol fluorene structures into the Poly(DL-lactide) chains resulted in an increased refractive index of the DL-BPF polymer, with a maximum value of 1.5276. This enhancement in refractive index can be advantageous for optical applications. However, it should be noted that the overall refractive index decreases with an increase in the molar ratio of DL-lactide monomers.

In the qualitative assessment of cytotoxicity based on morphology, it was observed that the 10DL-BPF polymer exhibited a cytotoxicity grade of 1, indicating low cytotoxicity. Furthermore, quantitative evaluation using the MTT assay revealed that the 100% 10DL-BPF extract exhibited a cell survival rate of 83.4%. These results demonstrate the good biocompatibility of the photocrosslinked 10DL-BPF polymer.

## Figures and Tables

**Table 1:** DL-BPF samples of molecular weight and state of matter gel fraction (%).

Polymer	DL:BPF ^a^	*M*_n_^c^ (g/mol)	*M*_nGPC_^b^ (g/mol)	*M*_wGPC_^b^ (g/mol)	*M*_w_/*M*_nGPC_^b^	Gel Residue (%)
5DL-BPF	5:1	1326	1252	1952	1.55	90.68
10DL-BPF	10:1	2046	2102	3313	1.57	93.55
15DL-BPF	15:1	2766	2856	4510	1.57	90.03
20DL-BPF	20:1	3486	3885	6165	1.58	90.94

^a^: DL-lactide-BPF = mol–mol, ^b^: Molecular weights determined by GPC calibrated with PS standards, GPC of Calibration curve, R = 0.9983. ^c^: Estimated molecular weight of DL-BPF: molecular weight of DL-lactide(144 g/mole) × DL-lactide molar number + molecular weight of BPF (606 g/mole).

**Table 2:** Refractive index of photocrosslinked DL-BPF.

Polymer	Refractive Index (nD)
BPF	1.6002
5DL-BPF	1.5276
10DL-BPF	1.5052
15DL-BPF	1.4946
20DL-BPF	1.4852

**Table 3:** Summary of DSC and TGA results.

Polymer	*T*_g,DSC_^A^ (°C)	*T*_g,DSC_^B^ (°C)	*T*_5%,TGA_^C^ (°C)	*T*_max1,TGA_^D^	*T*_max2,TGA_^D^	*T*_max3,TGA_^D^
PDLLA	41.3	–––	272.04	319.37	–––	–––
BPF	−13.1	70.0	213.60	–––	420.95	626.01
5DL-BPF	30.8	61.1	283.87	322.20	402.4	591.73
10DL-BPF	35.3	55.5	283.10	330.56	394.49	608.22
15DL-BPF	32.0	53.7	285.73	347.60	397.11	559.11
20DL-BPF	33.6	47.2	292.67	348.75	392.33	560.53

^A^: Pristine of DL-BPF. ^B^: Crosslinked of DL-BPF. ^C^: Thermal decomposition temperature of photocrosslinked DL-BPF polymer at 5% weight loss. ^D^: Maximum thermal decomposition temperature of photocrosslinked DL-BPF polymer.

## Data Availability

The data presented in this study can be made available upon request from the corresponding author. Please reach out to the corresponding author for access to the data.
